# When Art Moves the Eyes: A Behavioral and Eye-Tracking Study

**DOI:** 10.1371/journal.pone.0037285

**Published:** 2012-05-18

**Authors:** Davide Massaro, Federica Savazzi, Cinzia Di Dio, David Freedberg, Vittorio Gallese, Gabriella Gilli, Antonella Marchetti

**Affiliations:** 1 Research Unit on Theory of Mind, Department of Psychology, Università Cattolica del Sacro Cuore, Milan, Italy; 2 Research Unit on Psychology of the Art, Department of Psychology, Università Cattolica del Sacro Cuore, Milan, Italy; 3 Department of Neuroscience, University of Parma, Parma, Italy; 4 Department of Art History and Archaeology, Columbia University, New York, New York, United States of America; 5 The Italian Academy for Advanced Studies in America, Columbia University, New York, New York, United States of America; 6 IIT (Italian Institute of Technology) Brain Center for Social and Motor Cognition, Parma, Italy; Royal Holloway, University of London, United Kingdom

## Abstract

The aim of this study was to investigate, using eye-tracking technique, the influence of bottom-up and top-down processes on visual behavior while subjects, naïve to art criticism, were presented with representational paintings. Forty-two subjects viewed color and black and white paintings (Color) categorized as dynamic or static (Dynamism) (bottom-up processes). Half of the images represented natural environments and half human subjects (Content); all stimuli were displayed under aesthetic and movement judgment conditions (Task) (top-down processes). Results on gazing behavior showed that content-related top-down processes prevailed over low-level visually-driven bottom-up processes when a human subject is represented in the painting. On the contrary, bottom-up processes, mediated by low-level visual features, particularly affected gazing behavior when looking at nature-content images. We discuss our results proposing a reconsideration of the definition of content-related top-down processes in accordance with the concept of embodied simulation in art perception.

## Introduction

The debate on the definition of processes that contribute to the surfacing of an aesthetic experience is very controversial, partly because of the different weights assigned to the elements in the competition between “bottom-up” and “top-down” processes. Different theoretical frames emphasize one or the other process in the building up of an aesthetic experience. However it is most likely that, in looking at an artwork, an observer enters into a dialogue in which aesthetic experience emerges from the interaction between the two processes that operate at different levels of the viewer's experience [1]–[3]. In particular, top-down processes, classically recognized in factors such as content, cultural background and education, may interact and therefore affect bottom-up processes, generated by sensory-driven coding of external stimuli.

Given that aesthetic experience begins with a visual scan of the artwork, the multi-level interaction between sensory-driven bottom-up and top-down processes in aesthetic experience has been also studied exploring eye movement behavior [4], [5]. Pioneering investigations into visual exploratory behavior of paintings [6], [7] and subsequent studies on the informative details of an image [8], [9] revealed that observers focus their gaze on specific areas of the image, rather than in a random fashion. The areas receiving high densities of fixations were interpreted as cueing the observer's interest in informative elements of the image [10]. In fact, attention studies revealed that eye movements are an index of overt selection and, as a consequence, they are the expression of the relation between what is observed and its relevance to the viewer's interest [11]. In this respect, the analysis of the viewer's exploratory pattern and selection of salient visual aspects of the artwork can help shed light on the respective contribution of bottom-up and top-down processes in the first stages of aesthetic experience in the beholder.

The study of bottom-up processes involved in aesthetic experience has mainly focused on the analysis of image composition, i.e. the relation among visual features of an artwork [12]. In this respect, aesthetic experience appears to be influenced by factors such as contrast [13], balance [14], [15], maximum effects with a minimum of means [14] and symmetry [16]–[18]. Computational bottom-up models of visual exploration, using eye-tracking technique, have further identified the low-level properties responsible for drawing attention to specific areas of interest (salient regions of an image) [19]. Thus far, the identified contributors to visual saliency are contrast of luminance, curves, corners and occlusions as well as color, edges, lines and orientation [20].

There is evidence that low-level saliency measures, derived from a computational model (information theory), are also effective in capturing attention during aesthetic experience [21], [22]. For example, it has been shown that color may contribute to one's aesthetic experience [23] by enhancing the number of perceived elements within a composition, ultimately increasing image complexity. In fact, there is evidence that a moderate degree of complexity increases the aesthetic appeal of visual stimuli [24], [25]. Another factor that may contribute to visual saliency within a painting is dynamism. According to Arnheim [26] the recognition of some dynamic qualities of the image is one of the most important elements of the aesthetic experience. The way in which motion in art is represented was explored by a study showing that one of the few graphic invariants in Western visual art is that representing motion in garments. In these examples, motion perception is evoked by the adoption of specific features such as orientation, curvature and convergence of lines, which represent robust graphic elements that have survived, in the Western culture, across countries and centuries. The same effect can be gained also independently of contextual cues [27].

While the visual features that make up the structural composition of a representational artwork enhance the perceptual weight of the key elements within it (bottom-up processes), the goal of the visual exploration (task) may determine their informativeness for the viewer (top-down processes). As indicated above, top-down processes are influenced by a person's cultural background, education, degree of training in the arts, familiarity to and interest in a specific work of art [16], as well as by inter-individual differences [28]. Eye-movement studies have also indicated motivation and task requirement as top-down factors affecting aesthetic experience when viewing a painting [7], [29]. Platt and Glimcher [30] have shown that the reward macaque monkeys can expect from eye-movement responses modulates the activity of neurons within the oculomotor parietal area LIP. Rothkopf, Ballard, and Hayhoe [31] claimed that task requirements may be considered a good top-down predictor of gaze behavior. In fact they found that people involved in naturalistic virtual reality environments directed their gaze toward regions of the visual scene primarily on the basis of the task requirements. The evidence that eye movement patterns are affected by the cognitive task comes from studies in humans on high-level scene perception [10] as well as from visual aesthetic studies [5], [32]. Locher and colleagues [33], for example, showed that asking participants to assess either complexity or pleasantness of abstract dot patterns affected their visual exploratory behavior. Zangemeister and colleagues [32] also found that exploration pattern of the same abstract and realistic artworks changed as function of task requirements (no instruction, remember content features for a recall task or concentrate on artistic aspects of the artworks). In some other instances, investigations found only a moderate contribution of task-related top-down processes on gaze behavior during painting viewing. Wallraven et al. [21], for example, found that the scan paths of 20 participants, who looked at 275 artworks from different artistic styles under two different conditions (judging painting complexity, making aesthetic judgments), did not substantially change as a function of task-type. In fact, both tasks favored a global search strategy, although the spatial distribution of fixations was broader in the aesthetic judgment condition.

Additionally, the content of an artwork (for example a human portrait or the representation of a landscape) appears to influence human visual behavior in a top-down fashion. Although the structural composition of a painting may affect the perceptual weights of the most meaningful elements [5], [34], it has been also suggested that aesthetic experience associated with human content may operate in a specific fashion different from the mere structural features that characterize visual patterns lacking human forms. In this respect, semantic factors are shown to play an important role in preference ascription. In fact, image content appears to lead to greater divergence between factors, such as similarity and preference ratings, in representational works, and particularly in portraits, compared to artworks with poorer semantic values, such as abstract works [22]. One possible hypothesis of explanation of the relevance of semantic factors is the embodied theory of perception, which introduces a new element of aesthetic evaluation, namely, a multimodal notion of vision. Our visual perception of objects in the real world implies a lot more than the mere activation of our visual brain. Vision is always a multimodal enterprise, encompassing the activation of sensori-motor, viscero-motor and affect-related brain circuits. The discovery of mirror neurons [35], [36] and of a variety of mirroring mechanisms in our brain (for review, see [37]) demonstrated that the same neural structures activated by the actual execution of actions or by the subjective experience of emotions and sensations are also active when we see others acting or expressing the same emotions and sensations. These mirroring mechanisms have been interpreted as constituting a basic functional mechanism in social cognition, defined as embodied simulation [38], [39]. Embodied simulation is engaged also when actions, emotions and sensations are displayed as static images, as in the case of art works [40]. Mirroring mechanisms and embodied simulation, as suggested by Freedberg and Gallese [40] might empirically ground the fundamental role of empathy in aesthetic experience.

In the present study we used eye-tracking technique in the first stages of image scanning to investigate the contributions of bottom-up and top-down processes in the evaluation of aesthetic experience. The bottom-up processes under investigation were evoked by low-level features, namely color and dynamism; top-down processes were represented by task type and content of paintings. Eye movement behavior was studied while participants, naïve to art criticism, observed representational paintings in two experimental conditions: aesthetic judgment and movement judgment.

## Methods

### Participants

Forty-two Italian undergraduate students naïve to art criticism (22 female, 20 male, mean age = 22 S.D. = 3.95, range = 19–44) took part in this study. They gave their written informed consent to the experimental procedure. They did not present vision disorders that could interfere with the eye-tracking technique. Their participation was rewarded with a shopping voucher worth 20 euros. The study was approved by the Local Ethic Committee (Università Cattolica del Sacro Cuore, Milan).

### Visual Stimuli selection

One hundred stimuli were initially selected. They consisted of high-resolution digital versions of art paintings downloaded from different website collections. The stimuli were identified choosing artworks representing two main semantic categories: 50 human full-figure representations and 50 landscapes. Stimuli of these two groups were further categorized according to the level of represented movement for a total number of 4 sub-categories: 25 dynamic human images, 25 static human images, 25 dynamic nature images, and 25 static nature images. Three independent judges performed the categorization. The doubtful cases were collegially resolved. A second set of stimuli was obtained by digitally converting the colored paintings into black and white images. The color modification was performed using a photo editing computer program (Microsoft Office picture manager) by means of the standard tool incorporated in the software package. The issue of the decoloration of images is of great interest in the research on image digital manipulation. It is worth noting that several algorithms that aim to preserve the visual characteristics of color images have been developed (see for example, [41], [42]). However, there is not yet a strategy uniquely recognized as better than the others. The limits potentially linked to the decoloration strategy used should be kept in mind in evaluating any differences about the Color variable.

The aspect ratio of the paintings was preserved. Image sizes ranged from 448×880 to 519×797 pixels.

In order to select the 40 images considered being less familiar (not previously known), thirty-eight Italian volunteers (32 females, 6 males; age range = 20–61, mean age = 27.19, SD = 7.49), naïve to art criticism, were randomly assigned to the color or the black and white painting presentation and asked to express two different judgments about perceived familiarity and level of movement of each painting. Results confirmed our prior categorization of images into static and dynamic. Then, on the basis of familiarity judgment only, we selected the 40 images that obtained the lowest familiarity evaluation, equally distributed among the 4 sub-categories. Therefore, the following groups of images – both in color and black and white versions – were used for this study: 10 dynamic human images, 10 static human images, 10 dynamic nature images, and 10 static nature images (for a full detailed description of this procedure of selection and for more information about the paintings see Material S1 and Tables S1, S2, S3, S4). The size of these selected images ranged from 495×812 to 788×524 pixels.

### Procedure and Tasks

The stimuli were presented in two experimental tasks: aesthetic judgment (AJ) and movement judgment (MJ). The order of these two tasks was counterbalanced across participants. Eye-movements were recorded using an eye-tracking technique during both tasks.

Eighty stimuli (40 in the original color version and 40 in the modified black and white version) were presented on a computer screen in a randomized order. The presentation of the eye-tracking stimuli was created using the Tobii Studio 1.3 software (Tobii Technology AB). Participants were seated at a desk in a quiet room, at a distance of approximately 70 cm from the monitor. They were told that they would be shown a series of paintings on the computer monitor while their eye-position was recorded. Each trial began with the presentation of a central black cross on a white background for 1 second, followed by the presentation of the stimulus that lasted 3 seconds. Then, a task-related question about the aesthetic appreciation of the painting (AJ) or the movement perception (MJ) appeared. Participants were instructed to answer to the question on a 7-point Likert scale by using the PC mouse. The question was presented both at the beginning of each task and each time the answer was to be given. When the answer was given, the new trial started.

Each eye-tracker registration session lasted approximately 10 minutes. An initial calibration pattern was displayed to participants before running both the eye-tracker sessions (AJ and MJ tasks).

### Eye-Tracking data acquisition and model analysis

Eye position was recorded using a Tobii Eye-Tracker X120 set on the desk in front of the subject, between the subject and the monitor. The X120 Eye-Tracker is a stand-alone eye tracking unit that uses an infra-red based system for capturing reflections of the pupil and cornea in order to sample eye-position every 1/120 of a second. The system is accurate to less than 0.5 degrees.

**Figure 1:**
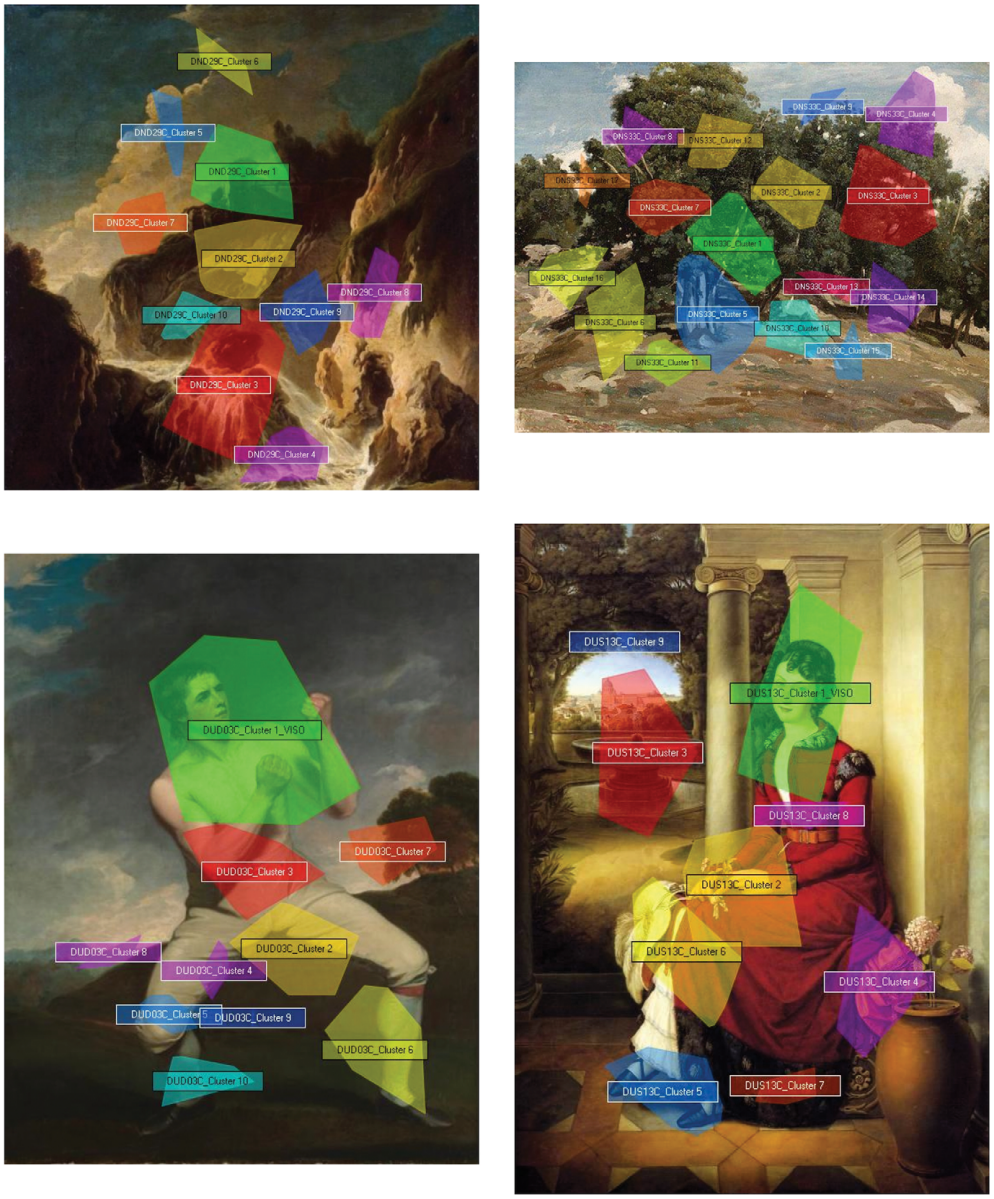
Examples of cluster distributions across color human and nature stimuli. On the left are dynamic images, on the right are static images.

Data were processed by the software through progressive aggregation levels in order to obtain a pattern of clusters, namely portions of the image with a high concentration of gaze data points. Clusters were automatically created by the software on the basis of the distance threshold that was set to 50 pixels as minimum distance between two different clusters (see figure 1). Tobii software uses the robust clustering algorithm suggested by Santella and DeCarlo [43] for eye movement data analysis. The cluster number represents the temporal order in which clusters were generated by the aggregation of fixations from each trial. Data were normalized with respect to the total area of images and of the size of clusters. The eye-movement indicators processed by the software (Tobii Studio 1.3) were fixations and observations. Fixations occur when a target feature of interest is positioned on the fovea for a variable period of time (averaging about 300 ms per fixation); observations occur each time a specific cluster is entered and exited. The data on these two eye-movements indicators were collected both in terms of number and duration. For the analysis of between-effects univariate GLM was used. As for the analysis of within- and between-effects fixed-effects ANOVA model was used. This model was chosen because robust and, therefore, able to provide very reliable results even with small sample sizes. It was preferred to a random-effects model (which would have allowed a stronger generalization of the results) since the latter, given the nature of our sample, would have increased the risk of biases in the computation of the model [44]–[46]. For the multiple comparisons the Sidak correction was applied.

#### Global pattern analysis

Analyses of eye-tracking data were firstly carried out within the total number of clusters formed in the paintings, corresponding to the sum of all clustered areas. For this purpose two indexes were created: 1) the total number of fixations per image, obtained by summing the number of fixations recorded for each cluster; 2) the mean duration of a fixation, obtained by dividing the total duration of fixations by the total number of fixations.

#### Cluster analysis

Gazing behavior within each cluster was analyzed. Since the minimum number of clusters built across all images was 4 (range 4–20), only the first 4 clusters (Regions of Interest, ROI) formed in temporal order of exploration were considered for the cluster analysis. The variables measured in this analysis are described in Table 1.

**Table 1:** Description of the variables used for the cluster analysis and the relative ascribed behavioral interpretation.

Measure	Description*	Interpretation
Time to first fixation.	Time in seconds from when the stimulus was shown until the start of the first fixation within the cluster.	Used within the first formed cluster, it indexes the attraction power/saliency of the content of that particular cluster. The more framed the image (expected content), the longer the time to first fixation.
Fixation number.	The number of the fixations within a cluster.	Richness of details.
Fixation duration.	The length of the fixation duration in seconds within a cluster.	Salience/relevance of the content.
Observation number and duration.	Number and duration of visits to a cluster.	Capacity of a cluster to capture attention; Salience/relevance of the content with respect to the other clusters//to the task.

* As reported in the Tobii Studio 1.X – User Manual v. 1.0 [62], pp. 82–86.

#### Latent Class Analysis

Latent class analysis (LCA) models containing one through four classes were fitted to the data using the 3.0 version of the Latent GOLD software [47]. LCA aims to define groups of subjects on the basis of the probability that each subject belongs to a specific group, investigating associations among a set of variables. This statistical method is particular useful and powerful because it does not rely on the traditional modeling assumptions and therefore it is less subject to biases associated with non-parametric data. The rationale for LCA is that the observed distance between subjects with respect to a specific set of variables is reduced by the identification of *n* classes, which maximize the internal homogeneity as well as the inter-class heterogeneity. Furthermore, unlike other techniques (for example K-means clustering), LCA provides various diagnostic tools in order to determine the optimal number of clusters. One of these is the Bayesan Information Criterion (BIC), based on the maximum likelihood function that allows selecting the best model among a finite set of models [48].

### Experimental aims

The present study aimed at answering the following research questions:

1. How do dynamism and color affect image exploration pattern (dynamic *vs.* static; color *vs.* black and white)?
2. Is there a specific exploration pattern associated with image content (human *vs.* nature)?
3. How do sensory-driven bottom-up and content-related top-down processes interact affecting the exploration pattern?
4. Is there a difference in exploration pattern between the types of task (aesthetic judgment *vs.* movement judgment) and is it correlated with the type of judgment expressed?

## Results

### Behavioral analysis

A 2×2×2 General Linear Model (GLM) analysis on the behavioral ratings with 2 levels of stimulus Content (human [H] *vs.* nature [N]), 2 levels of stimulus Dynamism (dynamic [D] *vs.* static [S]) and 2 levels of stimulus Color (color [C] *vs.* black & white [BW]) was carried out within the tasks of aesthetic judgment (AJ) and movement judgment (MJ) separately (see Table 2 and Table 3 for mean values and model statistical notations).

**Table 2:** Mean behavioral ratings per sub-category for AJ and MJ.

			Top-Down					
Judgments (1–7 Likert scale)			Movement (MJ)			Aesthetic (AJ)		
			Nature	Human	*Mean*	Nature	Human	*Mean*
**Bottom-Up**	**Black & White**	**Static**	2.67	1.75	*2.21*	3.51	3.40	*3.46*
		**Dynamic**	4.30	3.90	*4.10*	3.51	3.65	*3.58*
		*Mean*	*3.48*	*2.83*	*3.15*	*3.51*	*3.53*	*3.52*
	**Color**	**Static**	2.61	1.76	*2.19*	4.01	3.75	*3.88*
		**Dynamic**	4.42	4.04	*4.23*	4.46	4.02	*4.24*
		*Mean*	*3.51*	*2.90*	*3.21*	*4.24*	*3.89*	*4.06*
*Mean*			*3.50*	*2.86*	*3.18*	*3.87*	*3.71*	*3.79*

**Table 3:** GLM main effects and 2- and 3-ways interaction for Aesthetic and Movement ratings.

Indexes	Effect						
			*F*	*df*	*p*	*η^2^*	*δ*
**Aesthetic J**	**Dynamism**	D>S	10.453	1,41	<.01	.20	.88
	**Color**	C>BW	42.229	1,41	<.001	.51	.99
	Dynamism*Color		9.037	1,41	<.01	.18	.84
		DC>SC	16.703	1,41	<.001	.29	.98
	Content*Dynamism*Color		10.984	1,41	<.01	.21	.90
		HDC>HSC	4.590	1,41	<.05	.10	.55
		NDC>NSC	20.071	1,41	<.001	.33	.99
		HDBW>HSBW	5.160	1,41	<.05	.11	.60
**Movement J**	**Content**	H<N	20.275	1,41	<.001	.33	.99
	**Dynamism**	D>S	271.033	1,41	<.001	.87	.99
	Content*Dynamism		10.826	1,41	<.01	.21	.90
		HS<NS	41.969	1,41	<.001	.51	99
		HD<ND	4.586	1,41	<.05	.10	.56

**Figure 2:**
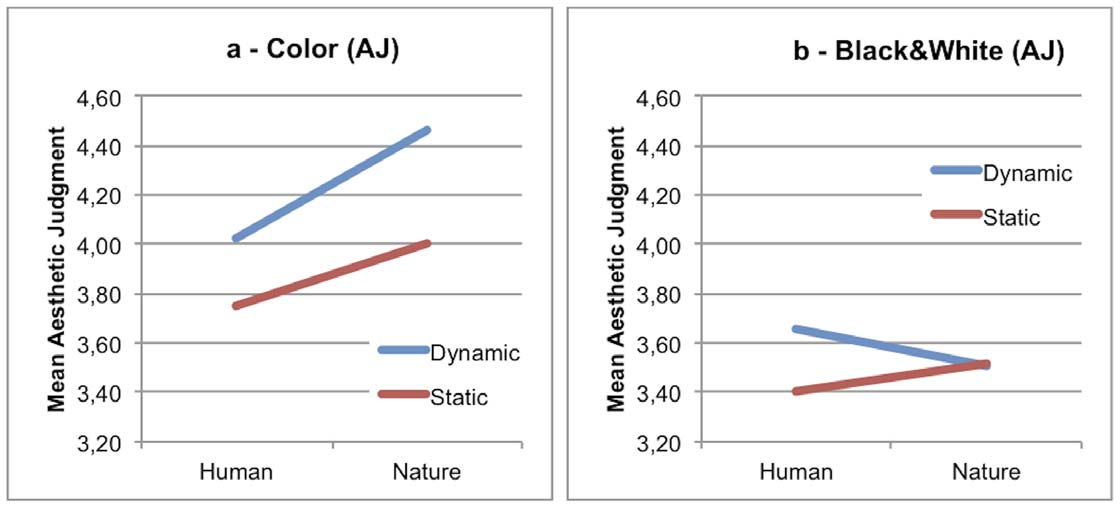
Aesthetic ratings in Content×Dynamism. On the left is the Color condition (a), on the right is the Black and White (b) condition.

As far as AJ task is concerned, results revealed a main effect of Dynamism (D>S) and a main effect of Color (C>BW). A significant interaction between Dynamism and Color was also found indicating that the significant difference between dynamic and static image ratings persisted only in the color condition (DC>SC). Additionally a 3 levels interaction was observed between Content, Color and Dynamism. More specifically, in the color condition (Figure 2a), human and nature images received a higher AJ in the dynamic condition than in the static condition (HDC>HSC; NDC>NSC). In the black and white condition (Figure 2b) only human dynamic images were preferred over human static images (HDBW>HSBW). What seems to emerge is a higher aesthetic appreciation for dynamic images than static ones. This appreciation seems to be influenced by the content of the picture. In the case of paintings representing nature, it remains high only in the presence of color that is a low-level characteristic; in the case of human figures, aesthetic appreciation may depend more on factors related to the content, namely high-level characteristics.

**Figure 3:**
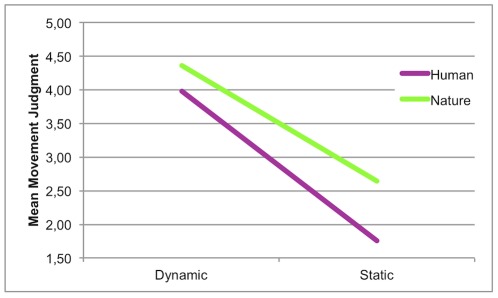
Movement ratings in Content×Dynamism.

**Figure 4:**
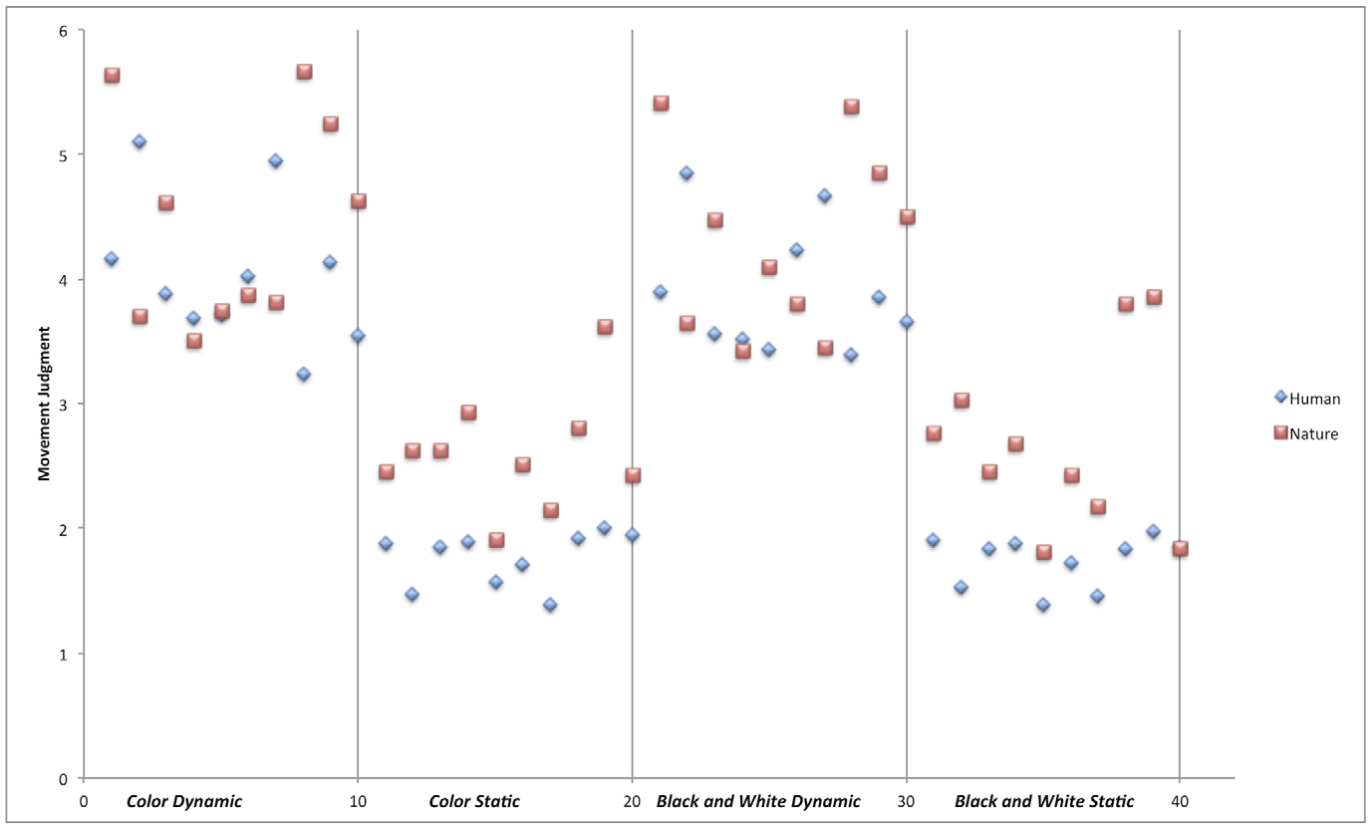
Movement ratings for human and nature images. Scattergram of movement rating for each stimulus.

With reference to MJ task, results showed a main effect of Content (H<N), a main effect of Dynamism (D>S) -confirming our prior stimulus selection– as well as an interaction between these 2 factors (Figure 3). Post-hoc analyses revealed that the magnitude of the difference between human and nature in static images (ΔM = .877; HS<NS) was greater than the magnitude of the difference between human and nature in dynamic images (ΔM = .388; HD<ND), although both of them were significant. The images representing nature are, on average, perceived as more dynamic than those representing human beings (Figure 4). This result could be explained with reference to a specific attraction exerted by the content of the paintings. Bodily driven mechanisms would mainly affect the exploration of human images, supporting a more precise and modulated perception of movement, whereas nature images would be mostly influenced by visual characteristics of the paintings.

### Eye-tracking global pattern analysis

#### Number of clusters

A univariate GLM analysis was conducted on the number of eye-fixation clusters as dependent variable with Content (human [H] *vs.* nature [N]), Dynamism (dynamic [D] *vs.* static [S]), Color (color [C] *vs.* black and white [BW]) and Judgment Task (aesthetic judgment [AJ] *vs.* movement judgment [MJ]) as independent variables (see Table 4 and Table 5 for mean values and model statistical notations). The movement and aesthetic ratings were not introduced in the present and subsequent models of eye tracking data because such ratings do not correlate with data of the exploration pattern, as shown and discussed below.

**Table 4:** Mean number of clusters per sub-category.

			Top-Down					
			Movement (MJ)			Aesthetic (AJ)		
			Nature	Human	*Mean*	Nature	Human	*Mean*
**Bottom-Up**	**Black & White**	**Static**	12.50	9.00	*10.75*	12.00	9.20	*10.60*
		**Dynamic**	9.70	9.20	*9.45*	11.20	8.90	*10.05*
		*Mean*	*11.10*	*9.10*	*10.10*	*11.60*	*9.05*	*10.33*
	**Color**	**Static**	12.40	8.70	*10.55*	13.10	9.30	*11.20*
		**Dynamic**	9.60	8.90	*9.25*	10.90	10.90	*10.90*
		*Mean*	*11.00*	*8.80*	*9.90*	*12.00*	*10.10*	*11.05*
*Mean*			*11.05*	*8.95*	*10.00*	*11.80*	*9.58*	*10.69*

**Table 5:** GLM main effects and 2-ways interaction for the number of clusters.

Index	Effect						
			*F*	*df*	*p*	*η^2^*	*δ*
**Nbr of Clusters**	**Content**	H<N	25.779	1,144	<.01	.15	.99
	**Dynamism**	D<S	4.101	1,144	<.05	.03	.52
	Content*Dynamism		9.138	1,144	<.01	.06	.85
		DN<SN	12.741	1,144	<.001	.08	.94
		HS<NS	32.806	1,144	<.001	.19	.99

Results revealed a main effect of Content (H<N) and a main effect of Dynamism (D<S). More specifically, the number of clusters was smaller in human than in nature images and in dynamic than in static images.

**Figure 5:**
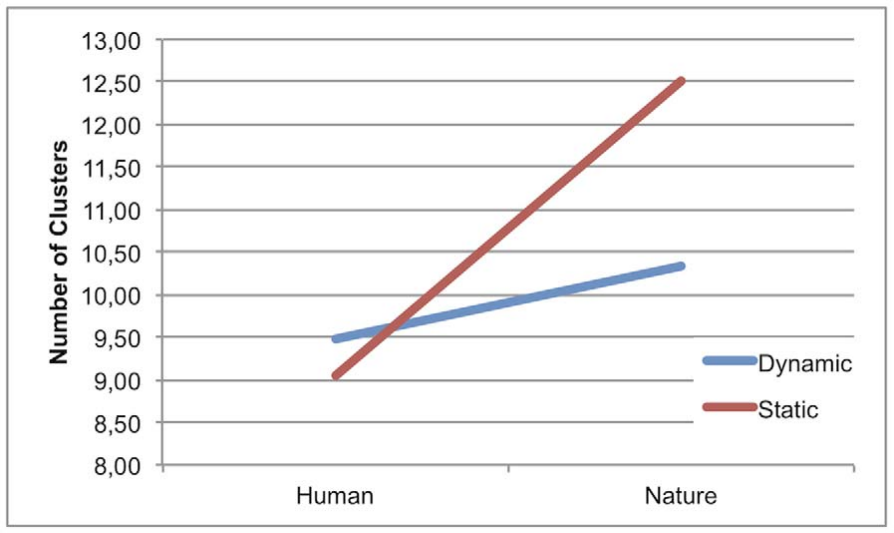
Number of clusters in Content×Dynamism.

An interaction between these 2 factors (Content and Dynamism) was also found (Figure 5). Post-hoc analyses revealed that dynamic images presented significantly fewer clusters than static images only in nature-content stimuli (DN<SN), whereas no significant differences in the number of clusters were found between dynamic and static images in the human-content stimuli. Furthermore, the effect of Content persisted only in the static condition (HS<NS). In fact, results did not show any significant difference in the number of clusters between human and nature condition in the dynamic images. No interaction effects were observed between any of the variables and Judgment Task-type. These data suggest a consistent influence of content-related processes on the overall exploratory pattern in terms of number of clusters. Images depicting a human content seem to hold defined elements of attraction (attractors) compared with nature images, in which attention appeared to be directed towards a greater and more variable number of potential attractors. The number of attractors in human-content paintings did not change as a function of dynamism; in these stimuli, in fact, attractors seem to be common in dynamic and static images, possibly sharing similar relevant features.

#### Total number of fixations and fixation mean duration

A 2×2×2×2 GLM was carried out on total number of fixations and mean duration of a fixation with 2 levels of stimulus Content (human [H] *vs.* nature [N]), 2 levels of stimulus Dynamism (dynamic [D] *vs.* static [S]), 2 levels of stimulus Color (color [C] *vs.* black and white [BW]) and 2 levels of Judgment Task (aesthetic judgment [AJ] *vs.* movement judgment [MJ]) (see Table 6, Table 7 and Table 8 for mean values and model statistical notations).

**Table 6:** Mean number of fixations on the total clustered area of images per sub-category.

			Top-Down					
			Movement (MJ)			Aesthetic (AJ)		
			Nature	Human	*Mean*	Nature	Human	*Mean*
**Bottom-Up**	**Black & White**	**Static**	7.62	5.25	*6.44*	7.56	5.23	*6.39*
		**Dynamic**	7.56	6.62	*7.09*	7.88	6.26	*7.07*
		*Mean*	*7.59*	*5.94*	*6.77*	*7.72*	*5.75*	*6.73*
	**Color**	**Static**	7.51	5.29	*6.40*	7.62	5.23	*6.43*
		**Dynamic**	7.68	6.65	*7.17*	8.01	6.93	*7.47*
		*Mean*	*7.60*	*5.97*	*6.78*	*7.82*	*6.08*	*6.95*
*Mean*			*7.60*	*5.95*	*6.78*	*7.77*	*5.91*	*6.84*

**Table 7:** Mean fixations duration (in seconds) on the total clustered area of the images per sub-category.

			Top-Down					
			Movement (MJ)			Aesthetic (AJ)		
			Nature	Human	*Mean*	Nature	Human	*Mean*
**Bottom-Up**	**Black & White**	**Static**	.29	.41	.*35*	.29	.41	.*35*
		**Dynamic**	.29	.34	.*32*	.28	.30	.*29*
		*Mean*	.*29*	.*37*	.*33*	.*29*	.*36*	.*32*
	**Color**	**Static**	.30	.42	.*36*	.28	.41	.*34*
		**Dynamic**	.30	.33	.*31*	.28	.30	.
		*Mean*	.*30*	.*37*	.*33*	.*28*	.*35*	.*32*
*Mean*			.*29*	.*37*	.*33*	.*28*	.*36*	.*32*

**Table 8:** GLM main effects and 2-ways interaction for the total number of fixations and fixation mean duration.

Indexes	Effect						
			*F*	*df*	*p*	*η^2^*	*δ*
**Total Number of eye fixations**	**Content**	H<N	291.813	1,41	<.001	.88	1.00
	**Dynamism**	S<D	256.800	1,41	<.001	.86	1.00
	Content*Dynamism		116.456	1,41	<.001	.74	1.00
		HS<HD	283.669	1,41	<.001	.87	1.00
		NS<ND	10.491	1,41	<.01	.20	.86
	Dynamism*Color		5.030	1,41	<.05	.11	.60
		CD>BWD	8.886	1,41	<.01	.18	.83
	Task*Color		5.711	1,41	<.05	.12	.65
		CAJ>BWAJ	10.112	1,41	<.01	.20	.87
**Mean duration of a single eye-fixation**	**Content**	H>N	125.805	1,41	<.001	.75	1.00
	**Dynamism**	S>D	156.831	1,41	<.001	.80	1.00
	Content*Dynamism		162.855	1,41	<.001	.80	1.00
		HS>HD	197.753	1,41	<.001	.83	1.00
	Dynamism*Task		14.402	1,41	<.001	.26	.96
		MJD>AJD	10.011	1,41	<.01	.20	.87

Results relative to the total number of eye-fixations revealed a main effect of Content (H<N) and a main effect of Dynamism (S<D). We found a lower number of fixations in the human-content as well as in static images than in nature and dynamic stimuli.

**Figure 6:**
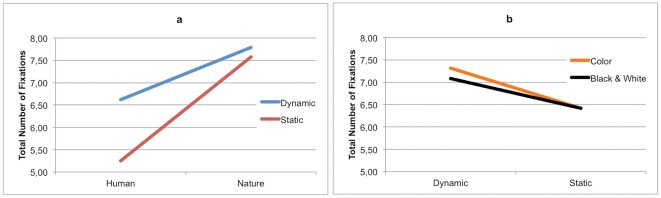
Total number of fixations in (a) Content×Dynamism and (b) Dynamism×Color.

Additionally, a significant interaction between Content and Dynamism was found. In human-content stimuli, static images counted a total number of fixations significantly lower than dynamic images (HS<HD; Figure 6a). Likewise, in nature-content stimuli, static images counted a total number of fixations significantly lower than dynamic images, which remained always higher than the corresponding values in the human-content condition (NS<ND). A significant interaction between Dynamism and Color was further found. The difference in the number of fixations between color and black and white images was observed only for dynamic stimuli, disappearing for static images (CD>BWD; Figure 6b).

Finally a significant interaction between Judgment Task and Color was found. During AJ task the number of fixations was significantly higher for the color images than for the black and white images (CAJ>BWAJ), whereas no difference was found in the number of fixations between color and black and white images during MJ task.

Considering the mean duration of a single-eye-fixation per image, results were complementary to those described above on the total number of fixations.

These first results about fixations corroborate the idea that human content guides the viewer's attention on a more limited number of attractors than nature content; however, human attractors are fixed for longer than nature attractors. Moreover, results show that dynamism and color have an enriching function of perceived details, supporting the fulfillment of the task.

### Eye-tracking cluster analysis

As specified in Methods session, analyses were carried out considering only the first 4 clusters (ROIs) formed in temporal order of exploration, which corresponded to the minimum number of clusters present in all images.

ROI analysis was carried out on the 4 first clusters using 2×2×2×2 GLM models with 2 levels of stimulus Content (human [H] *vs.* nature [N]), 2 levels of stimulus Dynamism (dynamic [D] *vs.* static [S]), 2 levels of stimulus Color (color [C] *vs.* black and white [BW]) and 2 levels of Judgment Task (aesthetic judgment [AJ] *vs.* movement judgment [MJ]).

#### Cluster size

Table 9 shows clusters size as a function of the percentage of area covered with respect to the total area of the image.

**Table 9:** Clusters size (%) in image representing human *vs.* nature content.

	Human				Nature			
	*Minimum*	*Maximum*	*Mean*	*SD*	*Minimum*	*Maximum*	*Mean*	*SD*
**Cluster 1**	.153	9.507	3.25	1.516	.933	10.881	4.415	2.051
**Cluster 2**	.277	6.7	3.268	1.267	.527	10.385	4.00	1.841
**Cluster 3**	.168	5.298	2.443	1.098	.157	8.844	2.911	1.960
**Cluster 4**	.099	5.396	1.918	1.151	.108	6.768	2.584	1.516

Results showed a main effect of Content (*F*_(4 141)_ = 14.773; *p*<.001, η^2^ = .30, δ = 1.00; H<N): ROIs extension was significantly smaller in human-content than in nature-content images, supporting the over mentioned idea that paintings representing human figures present highly meaningful and specific attractors.

#### Fixations and observations

ROI analysis was carried out within each of the 4 first ROIs considering the following indexes: time to first fixation, fixation number and duration, observation number and duration (Table 10).

**Table 10:** GLM main effects for fixations and observations on the first 3 ROIs.

Indexes	ROI	Content						Dynamism						Color						Task					
			*F*	*df*	*p*	*η^2^*	*δ*		*F*	*df*	*p*	*η^2^*	*δ*		*F*	*df*	*p*	*η^2^*	*δ*		*F*	*df*	*p*	*η^2^*	*δ*
**Fixations number**	*1*	H>N	70.66	1,41	<.001	.63	1.00	S>D	65.18	1,41	<.001	.61	1.00	BW>C	8.19	1,41	<.01	.17	.80	-					
	*2*	H>N	82.50	1,41	<.001	.67	1.00	D>S	92.28	1,41	<.001	.69	1.00	-						-					
	*3*	H>N	7.96	1,41	<.01	.16	.79	D>S	23.73	1,41	<.001	.37	1.00	-						-					
**Fixations duration**	*1*	H>N	246.33	1,41	<.001	.86	1.00	S>D	235.58	1,41	<.001	.85	1.00	BW>C	7.45	1,41	<.01	.15	.76	-					
	*2*	H>N	209.32	1,41	<.001	.84	1.00	D>S	74.93	1,41	<.001	.65	1.00	-						-					
	*3*	H>N	8.87	1,41	<.01	.18	.83	D>S	37.08	1,41	<.001	.48	1.00	C>BW	4.96	1,41	<.05	.11	.59	-					
**Observations number**	*1*	H>N	159.11	1,41	<.001	.80	1.00	S>D	47.06	1,41	<.001	.53	1.00	-						AJ>MJ	8.080	1,41	<.01	.17	.79
	*2*	H>N	87.70	1,41	<.001	.70	1.00	D>S	107.70	1,41	<.001	.72	1.00	-						-					
	*3*	H>N	10.47	1,41	<.01	.20	.89	D>S	14.58	1,41	<.001	.26	.96	-						AJ>MJ	7.036	1,141	<.05	.15	.74
**Observations duration**	*1*	H>N	283.25	1,41	<.001	.87	1.00	S>D	241.26	1,41	<.001	.85	1.00	BW>C	7.87	1,41	<.01	.16	.78	-					
	*2*	H>N	260.73	1,41	<.01	.86	1.00	D>S	83.81	1,41	<.001	.67	1.00	-						-					
	*3*	H>N	10.72	1,41	<.01	.21	.89	D>S	39.50	1,41	<.001	.49	1.00	C>BW	5.01	1,41	.<05	.11	.59	-					
**Time to first fixation**	*1*	H>N	32.475	1,41	<.001	.44	1.00	-						-						-					

As far as the time-to-first-fixation is concerned, in ROI 1 results showed a main effect of Content: the time necessary to enter into the first cluster was longer in human-content than in nature-content stimuli (H>N).

With regards to fixations and observations indexes in ROI 1, 2 and 3, results showed a main effect of Content: in all the three ROIs the fixations number and duration as well as the observations number were always higher in human-content than in nature-content images (H>N). Additionally, a main effect of Dynamism was also found for the first three ROIs. However, while in ROI 1 fixations and observations number and duration were higher in static images than in dynamic images (D<S), these effects reversed in ROI 2 and 3 (D>S). A similar trend was observed for the factor Color in ROI 1 and 3 only with respect to fixation and observation durations. In fact, in ROI 1 we found a longer duration of fixations and observations in black and white images than in color images (C<BW); this effect reversed in ROI 3 (C>BW). A higher number of fixations in black and white images than in color images was also found in ROI 1.

Finally, results revealed that in the considered clusters, Judgment Task affected observation number but not fixation indexes. Specifically, results showed a main effect in the observations number in ROIs 1 and 3 (AJ>MJ).

These principal effects confirm the attractive power of human-content images and highlight their informative strength, with a specific focus on the first three clusters. Furthermore, data show that, in the lack of the enriching effect of dynamism and color, attention focuses on ROI 1, probably because of its semantic value. Moreover, these meaningful portions of the image need to be re-explored for the ascription of an aesthetic evaluation.

Interaction analyses for each considered ROI and relative statistic values are summarized in Table 11. Among others, they show a significant interaction between Content and Dynamism. In ROI 1 the number and duration of fixations was higher in human static images than in human dynamic images (HS>HD), while in ROIs 2 and 3 these indexes were higher in human dynamic images than in human static images (HS<HD). A significant interaction was also found between Content and Color. Specifically, results revealed that, in ROI 1, black and white images received a higher number of fixations than color paintings only in nature-content (NC<NBW) and not in human-content images. Conversely, in ROI 2, the number of fixations and the duration of fixations were higher in color images than in black and white images only in human-content (HC>HBW) and not in nature-content stimuli. These results substantially confirmed the evidences emerged from principal effects, stressing further, on one side, the peculiarity of how images representing human figures drive the exploration pattern on specific portions of the image, on the other side, the role of color and dynamism as possible enhancer of paintings details.

**Table 11:** GLM 2-ways interaction for fixations on the first 3 ROIs.

Indexes	ROI	Content*Dynamism						Content*Color						Dynamism* Color					
			*F*	*df*	*p*	*η^2^*	*δ*		*F*	*df*	*p*	*η^2^*	*δ*		*F*	*df*	*p*	*η^2^*	*δ*
**Fixations number**	*1*	SH>DH	81.03	1,41	<.001	.66	1.00	BWN>CN	17.57	1,41	<.001	.30	.98	-					
	*2*	DH>SH	113.35	1,41	<.001	.73	1.00	CH>BWH	4.703	1,41	<.05	.10	.56	CD>BWD	9.44	1,41	<.01	.19	.85
	*3*	DH>SH	37.71	1,41	<.001	.48	1.00	-						-					
**Fixations duration**	*1*	SH>DH	278.17	1,41	<.001	.87	1.00	-						BWD>CD	49.04	1,41	<.001	.55	1.00
		-												-					
	*2*	DH>SH	100.92	1,41	<.001	.71	1.00	CH>BWH	6.86	1,41	<.05	.14	.73						
	*3*	DH>SH	58.35	1,41	<.001	.59	1.00	-						-					

		Judgment*Content						Judgment*Dynamism					
			*F*	*df*	*P*	*η^2^*	*δ*		*F*	*df*	*P*	*η^2^*	*δ*
**Fixations number**	*1*	HAJ>HMJ	8.104	1,41	<.01	.17	.79	SAJ>SMJ	10.612	1,41	<.001	.21	.89
	*2*	-						DMJ>DAJ	6.287	1,41	<.05	.13	.69
	*3*	-											
**Fixations duration**	*1*	-						SAJ>SMJ	36.803	1,41	<.001	.27	.96
		-						DMJ>DAJ	14.863	1,41	<.001	.47	1.00
	*2*	-						DMJ>DAJ	13.434	1,41	<.01	.25	.95
	*3*	-											

### Content Analysis and Latent Class Analysis (LCA)

Focusing only on human-content paintings, an analysis was carried out on the content of each ROI which was defined on the basis of a qualitative description of the portion of the body bounded by the ROI considered (face, limbs, trunk or mixed content – face+limbs or face+trunk –, not on human body). Results showed that the face area was the first clustered area (ROI 1) in the 61,3% of the cases; this value rose to 92.6% if also considering the content of ROI 2. Additionally, results revealed that the content mostly portrayed in the remaining 3 ROIs represented the limbs, on average, in 46% of the cases. See Table 12 for the percentage of fixations landed on these specific body parts.

**Table 12:** Percentage of fixations on the first 4 ROIs in human images.

Human figures	Static				Dynamic			
	*ROI 1*	*ROI 2*	*ROI 3*	*ROI 4*	*ROI 1*	*ROI 2*	*ROI 3*	*ROI 4*
**Face**	0,73	0,31	0,00	0,00	0,23	0,09	0,04	0,06
**Limbs**	0,06	0,49	0,59	0,25	0,16	0,27	0,42	0,46
**Trunk**	0,04	0,05	0,11	0,10	0,15	0,02	0,03	0,11
**Mixed content**	0,17	0,02	0,05	0,08	0,40	0,61	0,23	0,03
**Not human body**	0,00	0,12	0,25	0,56	0,06	0,02	0,28	0,35
**Total**	1	1	1	1	1	1	1	1

We carried out a latent class analysis (see Methods for details) based on the variables Dynamism (static *vs.* dynamic) and Judgment Task (aesthetic *vs.* movement) to identify the presence of content-driven exploration patterns considering the first four ROIs on human-content paintings. In other words, we intended to verify the presence of different explorative approaches focusing attention on the specific contents of the human body portrayed in the first four ROIs. In particular, LCA was fitted to the first four ROIs contents, which could vary between face, limbs, body and mixed contents (face+limbs or face+trunk).

In the first LCA the independent variable Dynamism (dynamic *vs.* static) was used as active covariate. Active covariates are predictors of the probability to belong to the latent classes. Considering the unexplained amount of the association among the variables (L^2^) and the explanative parsimony as selection criteria of the model, the best model was given by the 2-class model (L^2^ = 213.539 p<.01, Npar = 34, BIC = 850,96). The R^2^ values indicated that only the variance of the first two indicators (image clusters) was significantly explained by this 2-class model. In particular the model explained 22% and 31% of the variance respectively of the first and the second ROI. The covariate Dynamism significantly predicted the 2-class distinction. In fact, 73% of static images showed the predominance of face as content of the ROI 1, with a conditional probability (CP) equal to .71. This was followed by limbs as content of the ROI 2 (CP = .75). Eighty percent of dynamic images showed an homogeneous distribution of choice among limbs (CP = .28), body (CP = .31) and mixed content (CP = .29) for the ROI 1, and a predominant choice of mixed content (CP = .61) for the ROI 2. A LCA with the independent variable Judgment Task (aesthetic *vs.* movement judgment) as active covariate did not show any significant effect of this predictor.

LCA results show that – specifically for the exploration of human contents – in static images the semantic value of ROI 1 is consistently conveyed by face, whereas, in dynamic paintings, it is more equally represented by different portions of the body.

### Correlation Analysis

Correlations were carried out between aesthetic or movement behavioral ratings and eye-tracking variables. Significant correlations were found only with respect to clusters covering the face area in human images. In particular, correlations were observed between movement rating and number and duration of fixations (r = .309, *p*<.05; r = .324, *p*<.05, respectively) and between movement rating and duration of observation (r = .415, p<.01). The higher these indexes, the greater the movement evaluation.

## Discussion

The main aim of this study was to investigate the relationship between bottom-up and top-down processes while looking at representational paintings. Within this theoretical frame we specified variables pertaining to one or the other process. More specifically, we investigated exploration patterns during the observation of artworks presented in a color and in a black and white version (Color) and categorized as dynamic or static (Dynamism) (bottom-up processes). Images of paintings represented natural environments or human subjects (Content); they were displayed under aesthetic and movement judgment conditions (Task) (top-down processes). Our data are discussed against the classical approach to bottom-up and top-down processes and also propose alternative interpretations in the light of the results obtained. For simplicity, the effects of bottom-up processes (sensory-driven) on eye gazing behavior in relation to the top-down variables (content and task-type) are discussed in separate sections.

### Behavioral data

Behavioral results obtained in aesthetic judgment condition revealed that dynamic images were preferred to static images; likewise, color images were preferred to black and white images. However, interaction analyses showed that, when rating nature-content paintings only, aesthetic evaluation of dynamic images dropped appreciably in the absence of information about color. These results suggest that color might potentiate the aesthetic effect of dynamic images by possibly enriching the picture with perceptual details (increased image complexity). This idea is in line with Zellner et al. [25], who suggested that color –as a low-level saliency element– could increase the complexity of visual stimuli by enhancing the number of perceived elements, ultimately contributing to aesthetic experience [21]. This effect was not observed for human-content stimuli. In fact, preference for dynamic human images was not affected by information conveyed by color, suggesting that aesthetic evaluation of images depicting human subjects may be guided by content-related factors, which cannot be fully explained by low-level visual perceptual information only, as in the case of nature-content stimuli.

Additionally, the analysis of rating in movement judgment condition showed that nature images were on average recognized as more dynamic than human images. According to a classical perspective on movement perception, this result could be explained in terms of a more significant presence of low-level features in nature images that in human images. This visual information would elicit bottom-up processing of movement perception, highly affecting the formulation of a judgment [1]–[3]. However, this difference can be also explained in terms of content-related attractiveness to different aspects of the images. In fact, in nature-content paintings the dynamic character of the images was most likely affected by attention to low-level visually-driven bottom-up processes (e.g., color enhancing visual complexity); whereas, in human-content paintings, movement rating may have been affected by attraction to elements most possibly identified by bodily-driven simulation processes, that is, by the variety of sensory-motor resonance mechanisms induced by the observation of human bodies [40]. This mechanism would modulate the perception of movement in human images making it more detailed than that of nature images. This greater modulation would affect the rating variance, determining a lower average scoring for human movement than nature one.

This interpretation based on the concept of embodied simulation [38], [39] appears to be corroborated by data obtained from eye-tracking, as described in detail in the section to follow.

### Eye tracking data

#### Effect of bottom-up and content-related top-down processes

Eye-tracking results showed that static human-content images, on average, guided visual exploration on fewer precise areas than static nature images. The attraction exerted by human-content images was independent of dynamism, while nature-content stimuli attracted attention to few specific areas only in the case of dynamic images.

The lack of influence of dynamism while observing human-content paintings likely betrays the fact that a human body might imply an intrinsic and natural dynamism, evoking motor resonance in its beholder and causing, as earlier suggested, a more accurate perception of human than nature movement. This observation supports the hypothesis put forward in Graham et al. [22] where it is suggested that aesthetic experience, associated with human content, may rely on specific qualities of the artwork that are different from the structural features characterizing visual patterns lacking human forms. This result also supports the idea that, in the absence of a human figure, low-level visual features predominantly affect the visual scan path.

In fact, in static nature images, the greater number of clusters observed suggests that participants continued to explore the images in search of attractors. These latter, on the other hand, were more readily found in dynamic nature images, because of the specific low-level features employed in arts to represent motion [26].

What emerges from our data is that color and dynamism, at least for the paintings considered, appear to play an enriching function within bottom-up processes; whereas, within top-down bodily-driven processes, human content may show a stronger power than purely natural content. This interpretative frame finds further support if we consider the number of total fixations across paintings as well as cluster size. Dynamic and color images revealed a greater amount of perceived details than static and black and white paintings, as shown by a higher number of fixations. The smaller mean cluster size observed for human than nature paintings, on the other hand, indicates that attractors in human images captured attention on specific narrower areas than nature images and suggests, once more, that human images contained presumably more meaningful and informative bodily content elements than nature images. In fact, analysis of the first three clusters revealed more and longer fixations, as well as greater returns to these areas, in human than in nature paintings. They also confirmed that, in the lack of information about dynamism and color (static and black and white images), observer's attention was focused on the most salient part of the painting, namely cluster 1.

According to our hypothesis of embodied simulation, the human frame seems to automatically orient participants toward predetermined attractors, namely the presence of a human figure in the picture drives the search for parts of the body. This tendency may affect the time necessary to spatially identify the expected element. In fact, results revealed that the time used to make the first fixation into the first cluster was, on average, longer in human images, where the expected content is defined and framed, than in nature images, where the potential attractors may vary into a wider range of undefined elements. In other terms, in a picture depicting natural environments, any element may represent a potential attractor that requires inspection.

**Figure 7:**
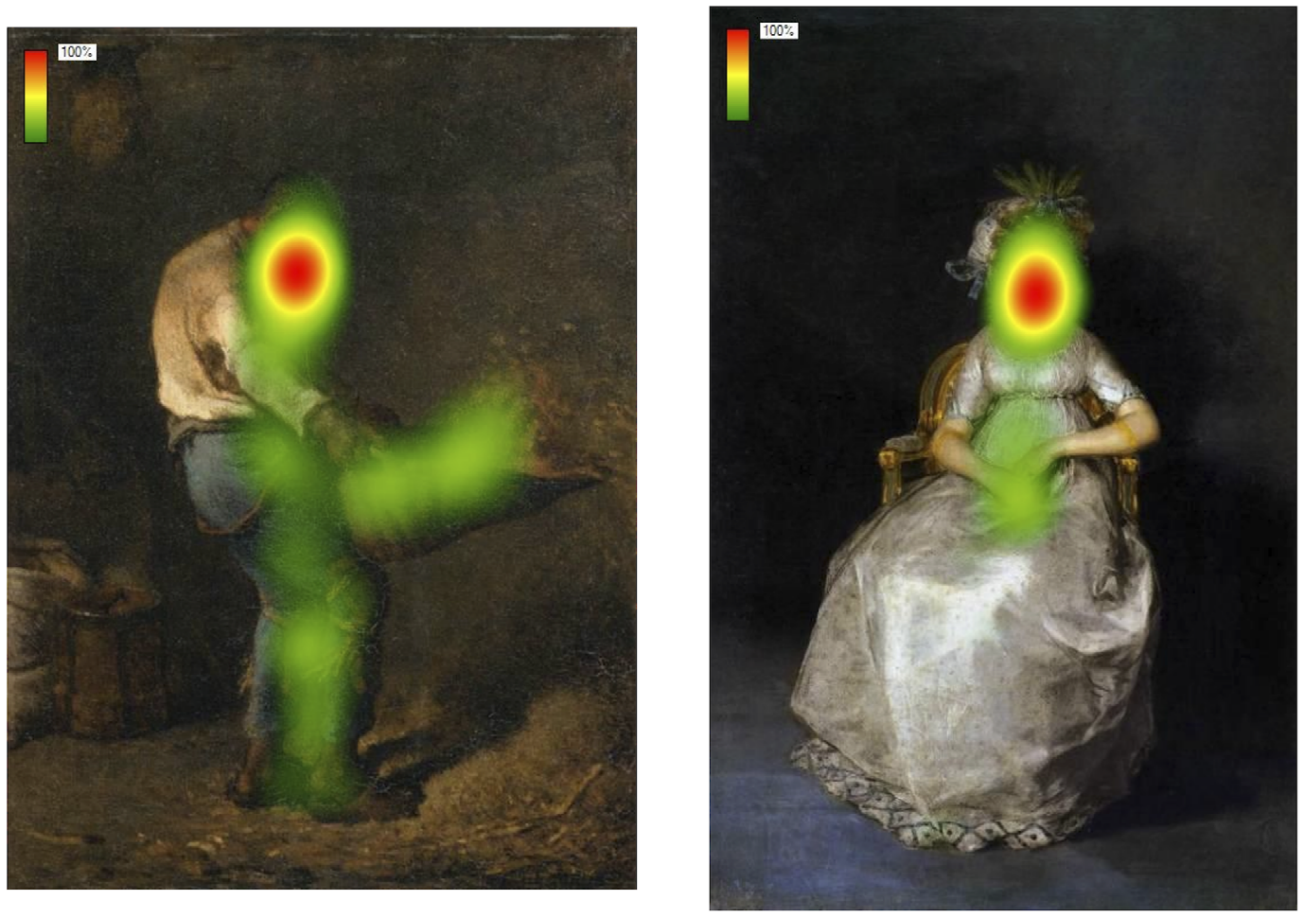
Heat map visualization of the gaze behavior for human color images. On the left is a dynamic image, on the right is a static image. The red gradient indicates portions of the image observed by the totality of the sample.

The interpretative framework arising from our results, thus far, gives a specific role to the human content – not found for the nature one – in the way it affects the aesthetic perception of paintings. This framework is further corroborated and extended by findings from Latent Class Analysis. Focusing only on human content images, it shows that in static images a strong attractor was face, while in dynamic images attention was equally spread out across different body parts. In the first case, the exploration pattern would be guided by the embodied simulation of sensations and emotions; in the second case it would be greatly influenced by the simulation of actions (see figure 7).

As for the face content, several studies showed that it is generally the first part of the body that is scanned in portraits [21] activating a configural visual encoding, instead of the more common analysis of individual features [22], [49], [50]. The importance of face was also shown in a study where eye-movements were recorded during the viewing of geometrical patterns that, in some instances, presented embedded faces. Results showed a variation in oculomotor behavior associated with the presence of face [51]. Attraction to face is particularly relevant because it represents an extremely important cue about a person's identity, health state, emotional state, attitude and gender, which are factors playing a crucial role when socially interacting with conspecifics [52]–[54]. It is interesting to observe that attraction to face, as highlighted by our findings, goes beyond the real social frame, it being triggered also when viewing humans represented in artworks.

As for the rest of the body, several studies suggested that also the human body might be a salient and powerful stimulus [55], [56]. For example, Calvo-Merino et al. [57] found that the perception of human bodies in dance postures, but not the vision of objects, activates specific motor areas. Body-sensitive areas contributed to aesthetic experience of dance perception as far as early analytical visual processing of body stimuli has a significant role in later aesthetic responses.

#### Effect of bottom-up and task-related top-down processes

Results relating to the number of clusters formed within the images showed that judgment tasks did not significantly affect the participants' behavior. Similarly, LCA showed that the attended areas (with respect to human-content only) were the same independently of whether the participants were assessing the aesthetics or the movement-expression of the paintings. Task-related top-down processes did not seem to have exerted a significant effect on overall exploration pattern.

However, results about more analytic eye-tracking indexes, indicated that the first clusters of the image, which were among the most salient in terms of represented content (see above), needed to be re-explored for the ascription of an aesthetic evaluation. In other terms, the identification of cues revealing motion were more readily recognized and processed during movement task than during aesthetic task, in which the identification of elements useful for an aesthetic assessment involved more explicit and evaluative processes.

In this respect, it should be added that we take an important component of aesthetic experience to be the response to perceptual objects consisting of the embodied simulation of emotions, sensations and actions, that the content of the object evokes in the beholder. Such experience is not necessarily confined to the appreciation of artworks, although this is grounded on it. In contrast, we conceive of aesthetic judgment as the explicit aesthetic rating of an object according to culturally and socially determined aesthetic canons. Aesthetic judgment represents the most cognitive aspect of the relation established with works of art and it answers to the question: “Is it beautiful?” [58], [59].

Deepening the interaction between bottom-up and task-related top-down processes, we found that color images were more explored than black and white images in aesthetic judgment task only. The capability of color to enrich the image of details, as already stressed in our discussion above, probably influenced participants' need for more fixations to evaluate the images aesthetically. Additionally, exploration was on average longer for dynamic images during movement judgment than during aesthetic judgment task, indicating, not surprisingly, that dynamic images were more significant in terms of task fulfillment. Correlation analyses between the various eye-tracking measures and the participants' behavioral ratings hardly produced any association. In other words, eye-gazing patterns were not predictive of either aesthetic or movement assessment of the observed stimuli. This lack of correlation is coherent with the results by Heidenreich and Turano [60] that did not show any significant link between participants' aesthetic judgments of the paintings and fixation durations or viewing time. On the whole, these data suggest that task-related top-down processes affected some specific components of the exploration pattern and that attraction exerted by sensory-driven bottom-up processes was functional to the fulfillment of the task.

Overall, our findings are subject to some limitations. Despite a considerable number of stimuli was used, it covers a limited portion of the artistic production available. Although the content categories analyzed (human and nature) are highly representative of what is commonly painted, they do not cover all categories of artistic content (for example, still life, human artifacts, etc.). Furthermore, some differences, although statistically significant, show a moderate magnitude and the type of analysis selected according to the characteristics of our sample, although robust, did not control for random effects.

### Concluding remarks

The relationship between top-down and bottom-up processes seems to stem from the salience of the content represented in the painting. We found that when represented content includes human subjects, content-related top-down processes prevail over low-level visually-driven bottom-up processes in guiding the observers' explorative pattern. On the other hand, when nature-content is represented, bottom-up processes, mediated by elements such as color, complexity and visual dynamism, appear to preferentially affect gazing behavior.

More specifically, when a human being is portrayed in a painting, gazing behavior is mostly focused on the human figure, independently of contextual elements also depicted in the image. In particular, attention is given to the face area, especially when ascribing an aesthetic judgment whereas dynamism ascription appears to be strongly guided by attention to features portraying actions. This evidence let us hypothesize that semantic content of artworks representing human body might evoke processes in the beholder that cannot be univocally explained with reference to classical socio-cultural factors (such as cultural background and education, see for example [1]–[3]), but that they also encompass the expression of embodiment, or, more specifically, of the feed-back signals fed by parieto-premotor sensory-motor circuits to oculo-motor and visual cortical areas.

In this respect, our results suggest an interpretation of the already described way of focusing attention in terms of embodied simulation: the face would elicit the simulation of emotions and sensations as well as the body would provoke the simulation of actions. This interpretation offers a new conceptualization of dynamism category that differs from the classical description of low level visually-driven bottom-up processes, yet recognized for nature-content paintings [21], [22]. More specifically, when a human subject is present in an image, the recognition of dynamism shifts from a visual decoding of perceptual elements (bottom-up process) to an embodied processing of the image semantics defined by the represented actions (bodily content-driven top-down process). In other terms, as suggested by Freedberg and Gallese [40], the hypothesis of embodied simulation would allow the identification of the emotions and the bodily engagement with the gestures, a pre-rational way to “make sense of the actions, emotions and sensations of others” (p. 198).

The question then arises of what determines dynamism perception in artworks representing nature. Is dynamism in paintings of natural scenes a sole effect of visual complexity, as our data suggest and, if so, in what terms is it coded? In terms of a possible physiological explanation, in which dynamism perception is associated with eye gazing variables, we hypothesized that, if perception of dynamism is a proprioceptive epiphenomenon elicited by eye-movements, there should be an association between number of fixations and movement judgment. Behavioral data obtained from movement judgment condition already indicated the lack of association between physiological measures and dynamism judgment in nature-content images. Additionally, analysis of physiological data alone showed that dynamic nature stimuli were characterized by a fewer number of clusters (narrow explorative behavior) than static stimuli and by equal number of fixations, suggesting that eye-movements did not affect the perception of dynamism in nature images. Perhaps, even when contemplating a waterfall, embodiment is relevant. As the German art historian Heinrich Wölfflin suggested [61] (p. 151) “…as human beings with a body that teaches us the nature of gravity, contraction, strength, and so on, we gather the experience that enables us to identify with the conditions of other forms”.

## Supporting Information

Material S1 **Preliminary study.** Method and procedure for eye-tracking stimuli selection.
(DOC)

Table S1 **Dynamic Human Paintings.** List of author, title, year and collection.
(DOC)

Table S2 **Static Human Paintings.** List of author, title, year and collection.
(DOC)

Table S3 **Dynamic Nature Paintings.** List of author, title, year and collection.
(DOC)

Table S4 **Static Nature Paintings.** List of author, title, year and collection.
(DOC)
