# Supplementary material for: When Art Moves the Eyes: A Behavioral and Eye-Tracking Study
Source: PLoS One. 2012 May 18;7(5):e37285. doi: 10.1371/journal.pone.0037285 (PMC3356266; doi:10.1371/journal.pone.0037285)
Supplement: Material S1 — Preliminary study. Method and procedure for eye-tracking stimuli selection. (DOC) [file pone.0037285.s001.doc]

**SUPPLEMENTARY MATERIAL OF THE PAPER:**

**When Art Moves the Eyes: A Behavioral and Eye-Tracking Study.**

Davide Massaro, Federica Savazzi, Cinzia Di Dio, David Freedberg, Vittorio Gallese, Gabriella Gilli, Antonella Marchetti

**PRELIMINARY BEHAVIORAL STUDY**

**Aims**

A behavioral study was performed to select images of representational paintings to use in the eye tracking experiment. This procedure aimed to support our categorization of images into static and dynamic (with reference to the level of represented movement) and to select the 40 images considered being less familiar (not previously known) by participants.

**Participants**

Thirty-eight Italian volunteers naïve to art criticism participated to the study (32 females, 6 males; age range = 20-61, mean age = 27.19, SD = 7.49). They were randomly assigned to one of the two experimental conditions: color and black and white painting presentations.

**Stimuli**

One hundred stimuli were initially selected. They consisted of high-resolution digital versions of art paintings downloaded from different website collections. The stimuli were identified choosing artworks representing two main semantic categories: 50 human full-figure representations and 50 outdoor landscapes. Stimuli of these two groups were further categorized according to the level of represented movement for a total number of 4 sub-categories: 25 dynamic human images, 25 static human images, 25 dynamic nature images, and 25 static nature images. Three independent judges performed the categorization. The doubtful cases were collegially resolved*.* A second set of stimuli was obtained by digitally converting the colored paintings into black and white images. The color modification was performed using a photo editing computer program (Microsoft Office picture manager). The aspect ratio of the paintings was preserved. Image sizes ranged from 448x880 to 519x797 pixels.

**Task**

The procedure was the same in the two experimental conditions. Volunteers were presented with images of paintings on a computer screen and were asked to express two different judgments about perceived familiarity and level of movement of each painting. Images were presented two times for each experimental condition (color vs. black and white). The two tasks were presented in the aforementioned fixed order. Each trial began with the presentation of a central white cross on a black background for 0.5 seconds, followed by the presentation of the stimulus that lasted 3 seconds. In the first task volunteers were asked to answer (yes or no) to the question “Is it the first time you see this painting?”. In the second task they answered the movement judgment question “In your opinion, how much does this painting convey sense of movement?”, using a 7-point Likert scale (1 = static to 7 = dynamic). Volunteers were instructed to answer to the task-question when a question mark appeared on the monitor. They had 4 seconds to give their answer. Images were randomly presented. The full experimental session lasted for maximum 28 minutes.

**Results**

A 2x2x2 General Linear Model (GLM) with the movement rating as dependent variable and 2 levels of stimulus Content (human *vs*. nature) and Dynamism (dynamic *vs*. static) as within-subjects independent variables, and 2 levels of stimulus Color (color vs. black and white) as between-subjects independent variable was carried out.

Results revealed a main effect of Dynamism (*F*_(1 36)_ = 368.124; *p* < .001, *η*^2^ = .91, *δ* = 1.00), showing higher movement rates for dynamic than static images. These findings confirmed our categorization of images into static and dynamic.

Then, on the basis of familiarity judgment only, we selected the 10 less known images, both in color and black and white version, for each typology (Content x Dynamism). The 40 images selected were those that obtained the lowest familiarity evaluation. See Tables S1, S2, S3, S4 for the list of selected artworks.
