# Supplementary material for: When Art Moves the Eyes: A Behavioral and Eye-Tracking Study
Source: PLoS One. 2012 May 18;7(5):e37285. doi: 10.1371/journal.pone.0037285 (PMC3356266; doi:10.1371/journal.pone.0037285)
Supplement: Table S1 — Dynamic Human Paintings. List of author, title, year and collection. (DOC) [file pone.0037285.s002.doc]

Table S1. Dynamic Human Paintings

| **Title** | **Artist** | **Year** | **Collection** |
| --- | --- | --- | --- |
| The Flea | Crespi, Giuseppe Maria | 1707-1709 | Galleria degli Uffizi, Florence |
| Miss La La at the Cirque Fernando | Degas, Edgar | 1879 | National Gallery, London |
| Richard Humphreys, the Boxer | Hoppner, John | XVIII sec. | The Metropolitan Museum of Art, New York |
| The Morning Toilet | Steen, Jan | c. 1665 | Rijksmuseum, Amsterdam |
| At the Mirror | Kersting, Georg Friedrich | 1827 | Kunsthalle, Kiel |
| The Evening | Leicher, Felix Ivo | 1780s | Museum of Fine Arts, Budapest |
| The Winnower | Millet, Jean- François | 1848 | Musée du Louvre, Paris |
| La Japonaise | Monet, Claude | 1876 | Museum of Fine Arts, Boston |
| Apollo | Dossi, Dosso | 1524 | Galleria Borghese, Rome |
| Une jeune fille s'apprêtant à orner la statue de l'Amour d'une guirlande de fleurs | Roslin, Alexandre | 1783 | Musée du Louvre, Paris |
