# Supplementary material for: When Art Moves the Eyes: A Behavioral and Eye-Tracking Study
Source: PLoS One. 2012 May 18;7(5):e37285. doi: 10.1371/journal.pone.0037285 (PMC3356266; doi:10.1371/journal.pone.0037285)
Supplement: Table S2 — Static Human Paintings. List of author, title, year and collection. (DOC) [file pone.0037285.s003.doc]

Table S2. Static Human Paintings

| **Title** | **Artist** | **Year** | **Collection** |
| --- | --- | --- | --- |
| Portrait of an Artist in His Studio | Géricault, Théodore | c. 1820 | Musée du Louvre, Paris |
| The Countess of Chinchón | Goya Y Lucientes, Francisco De | 1800 | Museo del Prado, Madrid |
| Marchesa Florenzi | Hess, Heinrich Maria Von | 1828 | Neue Pinakothek, Munich |
| Old Woman Dozing | Maes, Nicolaes | 1656 | Musées Royaux des Beaux-Arts, Brussels |
| The Empress Doña Margarita de Austria in Mourning Dress | Mazo, Juan Bautista Martinez Del | 1666 | Museo del Prado, Madrid |
| Ferdinand IV, King of Naples | Mengs, Anton Raphael | 1760 | Museo del Prado, Madrid |
| Old Woman Seated | Puga, Antonio | XVII sec. | Museo del Prado, Madrid |
| Portrait of Heinrike Dannecker | Schick, Christian Gottlieb | 1802 | Staatsgalerie, Stuttgart |
| Portrait of Wilhelmine Cotta | Schick, Christian Gottlieb | 1802 | Staatsgalerie, Stuttgart |
| Magdalene | Caravaggio | 1596-1597 | Galleria Doria-Pamphili, Rome |
