# Supplementary material for: When Art Moves the Eyes: A Behavioral and Eye-Tracking Study
Source: PLoS One. 2012 May 18;7(5):e37285. doi: 10.1371/journal.pone.0037285 (PMC3356266; doi:10.1371/journal.pone.0037285)
Supplement: Table S3 — Dynamic Nature Paintings. List of author, title, year and collection. (DOC) [file pone.0037285.s004.doc]

Table S3. Dynamic Nature Paintings

| **Title** | **Artist** | **Year** | **Collection** |
| --- | --- | --- | --- |
| Le torrent (Tivoli) | Michallon, Achille-Etna | 1818-1821 | Musée du Louvre, Paris |
| Weymouth Bay, with Jordan Hill | Constable, John | 1816 | National Gallery, London |
| Cloud Study with Horizon | Dahl, Johan Christian Clausen | 1832 | Nationalgalerie, Berlin |
| Scogliera a Sestri Levante | Gignous, Eugenio | 1890 | Private Collection |
| Drifting Clouds | Friedrich, Caspar David | c. 1820 | Kunsthalle, Hamburg |
| Fog in the Elbe Valley | Friedrich, Caspar David | 1821 | Nationalgalerie, Berlin |
| The North Sea in Moonlight | Friedrich, Caspar David | 1823-1824 | National Gallery, Prague |
| The Waterfalls at Terni | Hackert, Jacob Philipp | 1779 | Private Collection |
| Landscape with a Waterfall | Roos, Philipp Peter | XVII sec. | The Hermitage, St. Petersburg |
| Rome: Study of Clouds | Valenciennes, Pierre-Henri De | 1780s | Musée du Louvre, Paris |
