# Supplementary material for: When Art Moves the Eyes: A Behavioral and Eye-Tracking Study
Source: PLoS One. 2012 May 18;7(5):e37285. doi: 10.1371/journal.pone.0037285 (PMC3356266; doi:10.1371/journal.pone.0037285)
Supplement: Table S4 — Static Nature Paintings. List of author, title, year and collection. (DOC) [file pone.0037285.s005.doc]

Table S4. Static Nature Paintings

| **Title** | **Artist** | **Year** | **Collection** |
| --- | --- | --- | --- |
| Aurore, novembre | Ballot, Clémentine | 1915 | Centre Pompidou, Paris |
| Campagna Landscape | Böcklin, Arnold | 1857-1858 | Nationalgalerie, Berlin |
| Fontainebleau: Oak Trees at Bas-Bréau | Corot, Camille | c. 1832 | The Metropolitan Museum of Art, New York |
| George Sand's Garden at Nohant | Delacroix, Eugène | 1840s | The Metropolitan Museum of Art, New York |
| Bohemian Landscape with Mount Milleschauer | Friedrich, Caspar David | 1808 | Gemäldegalerie, Dresden |
| The Watzmann | Friedrich, Caspar David | 1824-1825 | Nationalgalerie, Berlin |
| The Oaktree in the Snow | Friedrich, Caspar David | 1829 | Nationalgalerie, Berlin |
| Marina di Castiglioncello (studio) | Sernesi, Raffaello | c. 1864 | Private Collection |
| Rocky Crags at l'Estaque | Renoir, Pierre-Auguste | 1882 | Private Collection |
| Le Bas-Bréau, à Chailly | Leprince, Robert-Léopold | 1825 | Musée du Louvre, Paris |
